# Associations Between Delivery Modes, Birth Outcomes and Offspring Anxiety Disorders in a Population-Based Birth Cohort of Children and Adolescents

**DOI:** 10.3389/fpsyt.2022.917299

**Published:** 2022-07-13

**Authors:** Tiia Ståhlberg, Subina Upadhyaya, Päivi Polo-Kantola, Prakash Khanal, Terhi Luntamo, Susanna Hinkka-Yli-Salomäki, Andre Sourander

**Affiliations:** ^1^Research Center for Child Psychiatry, INVEST Flagship, University of Turku, Turku, Finland; ^2^Department of Obstetrics and Gynecology, Turku University Hospital and University of Turku, Turku, Finland; ^3^Department of Child Psychiatry, Turku University Hospital, Turku, Finland

**Keywords:** cesarean section, perinatal care, epidemiology, anxiety disorders, child psychiatry, adolescent psychiatry, registries

## Abstract

**Objective:**

Mode of delivery and well-being markers for newborn infants have been associated with later psychiatric problems in children and adolescents. However, only few studies have examined the association between birth outcomes and anxiety disorders and the results have been contradictory.

**Methods:**

This study was a Finnish population-based register study, which comprised 22,181 children and adolescents with anxiety disorders and 74,726 controls. Three national registers were used to collect the data on exposures, confounders and outcomes. Mode of delivery, the 1-min Apgar score, umbilical artery pH and neonatal monitoring were studied as exposure variables for anxiety disorders and for specific anxiety disorders. Conditional logistic regression was used to examine these associations.

**Results:**

Unplanned and planned cesarean sections increased the odds for anxiety disorders in children and adolescents (adjusted OR 1.08, 95% CI 1.02–1.15 and aOR 1.12, 95% CI 1.05–1.19, respectively). After an additional adjustment for maternal diagnoses, unplanned cesarean sections remained statistically significant (aOR 1.11, 95% CI 1.04–1.18). For specific anxiety disorders, planned cesarean sections and the need for neonatal monitoring increased the odds for specific phobia (aOR 1.21, 95% CI 1.01–1.44 and aOR 1.28, 95% CI 1.07–1.52, respectively).

**Conclusions:**

Birth by cesarean section increased the odds for later anxiety disorders in children and adolescents and unplanned cesarean sections showed an independent association. Further studies are needed to examine the mechanisms behind these associations.

## Introduction

Anxiety disorders are the most common psychiatric disorders (1) and among the earliest to manifest during life (2). They may be prolonged, lead to secondary diagnoses and cause notable impairment (1, 3). The etiological risk factors include some genetic and childhood environmental factors (4), but the role of perinatal factors is less clear, especially those during birth. Obstetrical adversities have been associated with various neurodevelopmental and psychiatric disorders (5–8). For example, perinatal hypoxemia, ischemia, infections, inflammation and stress may alter neurodevelopment and the hypothalamus-pituitary-adrenal axis (9, 10). These serve as potential mechanisms in the association between perinatal factors and later psychiatric problems (11). Maternal stress, due to perinatal adversities, may also have a negative impact on parenting styles and interaction, which could contribute to anxiety disorders in their offspring (12).

Large register-based studies are ideal for studying perinatal factors, because they can include whole birth cohorts, detailed confounders and also eliminate recall bias. Five register-based studies have examined birth outcomes and anxiety disorders (13–17). Four found that cesarean sections increased the risk for anxiety disorders (13–16). However, the associations with planned and unplanned cesarean sections disappeared in one study when they were adjusted for specific indications for cesarean section (13). The fifth did not find any associations (17). Low Apgar scores increased the risk for anxiety disorders in one study (17), but not in two other studies (15, 16) (Supplementary Table 1, available online). The definition of anxiety disorders varied, as four studies also included obsessive-compulsive disorder or stress-related disorders (13–15, 17). These are no longer included in the anxiety disorder category in the fifth version of Diagnostic and Statistical Manual of Mental disorders, as they stem from a slightly different etiology (18). It is notable that only two studies included childhood anxiety disorders (15, 17). In addition, planned and unplanned cesarean sections were only separated in one study (13). No register studies examined specific anxiety disorders separately. One small cohort study found that treatment in a neonatal intensive care unit (NICU) was associated with specific phobias (19) and two identified it to associate with separation anxiety disorders (19, 20).

The main aim of this study was to examine the associations between mode of delivery, birth outcomes and anxiety disorders using a large nationwide register-sample of children and adolescents. The exposure variables were mode of delivery, Apgar score, umbilical artery pH and neonatal monitoring and the confounders included various other perinatal events. The second aim was to examine the associations between mode of delivery, birth outcomes and specific anxiety disorders.

## Methods

**Figure 1:**
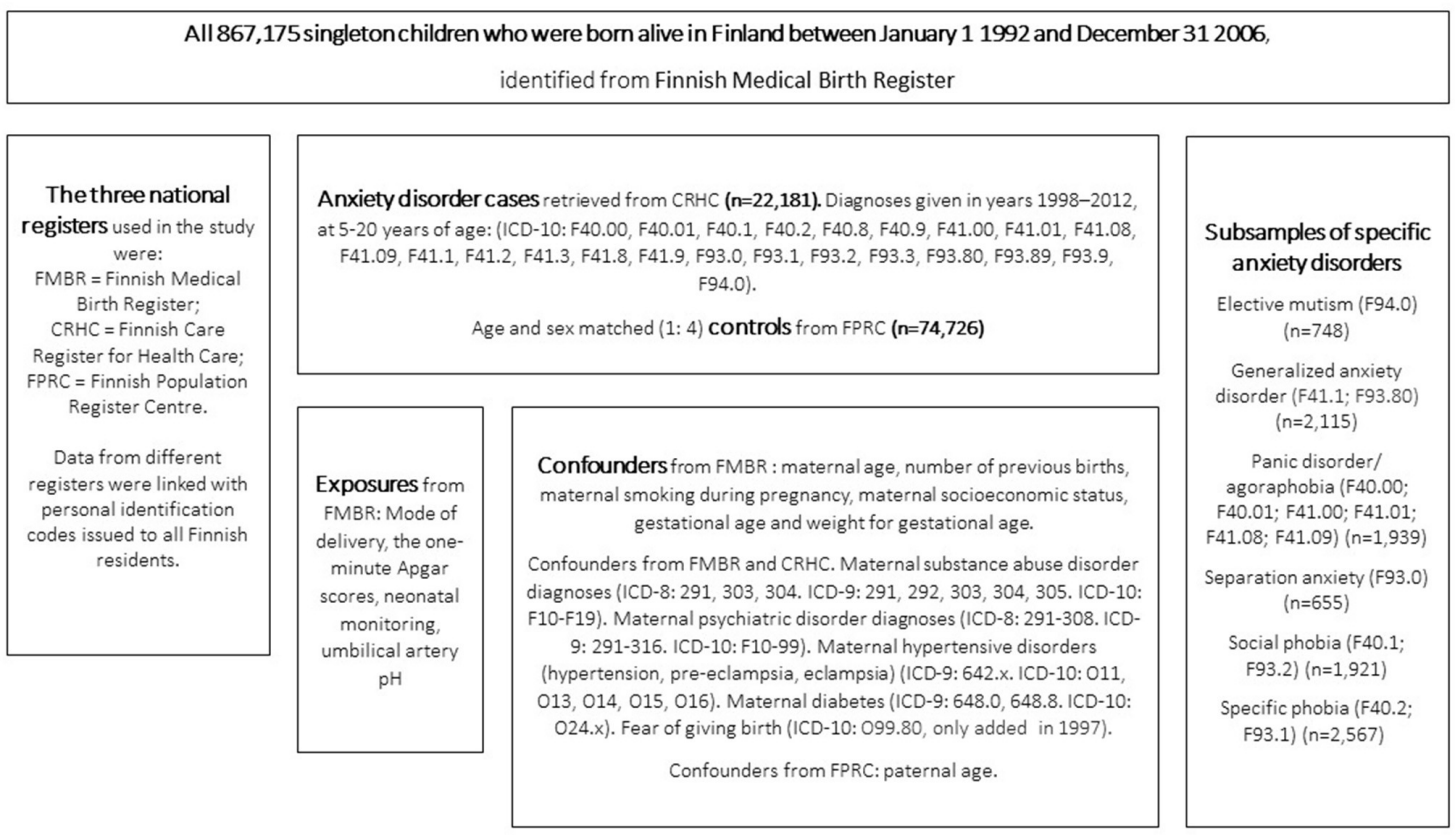
Study design.

This register-based study was part of the Finnish Prenatal Study of Anxiety, an ongoing nested case-control study that uses data from three national registers (Figure 1). The use of this data was approved by the Finnish Data Protection Ombudsman.

### Data Sources

The data from each register are described in Figure 1. The Finnish Care Register for Health Care was established in 1969 (21) and provides diagnostic information from specialized healthcare services. The Finnish Medical Birth Register, established in 1987, contains a great variety of prenatal and perinatal information about infants born alive or stillborn in Finland, with gestational ages of ≥22 weeks or birth weights of ≥500 grams (22). The Finnish Population Register Centre provides information on permanent Finnish residents. The data on these registers can be linked by the personal identification codes, which are unique codes issued to all Finnish residents. The linkage enabled to collect the data accurately for exposures and confounders for each case and control subject of this study.

### Cases and Controls

The study sample was a birth cohort of all 867,175 singletons born alive in Finland in 1992–2006. We identified those diagnosed with an anxiety disorder between 1998 and 2012. The births were collected from the Finnish Medical Birth Register and the diagnoses from the Finnish Care Register for Health Care. Four sex and age matched controls without anxiety disorders were identified for each case. Controls were excluded if they received an anxiety disorder diagnosis during the 2012–2016 follow-up period or if they had missing data. Cases and controls with severe of profound intellectual disabilities, with the International Classification of Diseases, Tenth Revision (ICD-10) codes of F72 and F73 (23), were excluded. The final cohort comprised 22,181 cases with an anxiety disorder diagnosis and 74,726 controls.

### Outcomes

The outcome was any anxiety disorder diagnosis in the Finnish Care Register for Health Care from 1998–2012. The Register has provided inpatient data from 1969, but only started recording outpatient data in 1998. Physicians used the ICD-10 anxiety disorder diagnoses (23). The incident diagnoses for the cases were included from the year the subjects turned 6 years. Following ICD-10 anxiety disorder diagnoses were included: F40, F41, F93, and F94.0. Additionally, the following specific anxiety disorders were examined separately: elective mutism (*n* = 748), generalized anxiety disorder (*n* = 2,115), panic disorder and/or agoraphobia (*n* = 1,939), separation anxiety disorder (*n* = 655), social phobia (*n* = 1,921), and specific phobia (*n* = 2,567). Detailed diagnostic information is presented in Figure 1.

### Exposure Variables

Four exposure variables were selected from the Birth Register: mode of delivery, the 1-min Apgar score, umbilical artery pH and neonatal monitoring. There were four categories for delivery. Vaginal deliveries were split into spontaneous, unassisted vaginal cephalic deliveries and other types, which included induced delivery, vacuum or forceps and breech. Cesarean deliveries were split into planned and unplanned, which included urgent, emergency or other. The 1-min Apgar score was categorized as ≥7 or <7. Low Apgar score indicates disturbances in color, pulse, reflexes, muscle tone or respiratory function (24). The more feasible 5-min Apgar score was not available bit for 2.9% of the cases and therefore not utilizable. The umbilical artery pH was both a categorical (≥7.15 or <7.15) and continuous variable. Low pH values indicate asphyxia (25). Routinely measuring umbilical artery pH only began during the study period and was only available for 43.1% of the sample. Neonatal monitoring was defined as “no” referring to normal neonatal treatment or “yes” referring to enhanced monitoring, admitted by a pediatrician and carried out in a NICU or maternal postpartum ward.

### Confounders

The confounder variables were based on our previous studies (6, 7, 26) and literature (15–17) and obtained from the three national registers (Figure 1). These were tested for relevance before being added to the multivariate model. They were: maternal and paternal age, number of previous births, maternal and paternal psychiatric disorders (maternal substance use disorders excluded), maternal substance abuse, smoking during pregnancy and socioeconomic class (SES), gestational age and weight for gestational age. Three additional cofounders were chosen for the supplementary mode of delivery analysis, based on the literature and availability in the registers. These were: maternal hypertensive disorders (27, 28), diabetes (29, 30) and fear of giving birth (31, 32).

The missing data for the confounders was relatively small. Maternal SES was missing for 4.8% of the subjects and handled as a missing category. Smoking was missing for 2.2% and paternal age for 1.2% (Supplementary Table 2). There was no collinearity between maternal SES and smoking. The ICD-10-diagnosis O99.80, fear of giving birth was only established in 1997 (26, 32) and therefore not available for the oldest subjects.

### Statistical Methods

The odds ratios (OR) and the 95% confidence intervals (95% CI) were obtained by fitting conditional logistic regression models for matched sets. The chi-square test was used to examine the association between each potential confounder and each exposure in the controls. We also used conditional logistic regression to test the associations between possible confounders and diagnosed anxiety disorders in the case-control model. Confounders were included in the final conditional logistic regression model if both of these analyses yielded significant associations (*p* < 0.05) with at least one exposure. We included all exposures with significant associations in the unadjusted model. An additional conditional logistic regression model was created for mode of delivery, with additional confounders that indicate the need for a cesarean section. These models were used also to analyze specific anxiety disorders. Statistical significance was *p* < 0.05. SAS version 9.4 was used for the statistical analyses (SAS Institute, Inc., Cary, NC, USA).

## Results

Table 1 describes the frequencies for the 22,181 cases (56.4% female) and 74,726 controls by each exposure and confounder. The mean age for anxiety disorder diagnoses was 12.7 ± 3.7 years (range 5–20 years).

**Table 1:** Associations between exposures and anxiety disorders in the unadjusted and adjusted analyses.

**Exposure variable**	**Cases *n* (%)** ** (*n =* 22,181)**	**Controls *n* (%)** ** (*n =* 74,726)**	**Unadjusted analyses, OR** ** (95% CI)**	***p*-value**	**Adjusted analyses Model 1,** ** OR (95% CI)**	***p*-value**	**Adjusted analyses Model 2, OR** ** (95% CI)**	***p*-value**
**Mode of delivery**								
*Spontaneous vaginal cephalic*	17,132 (77.3)	59,147 (79.2)	Reference	Ref	Reference	Ref	Reference	Ref
*Vaginal other*	1,279 (5.8)	4,283 (5.7)	1.04 (0.98 to 1.11)	0.23	1.04 (0.97 to 1.12)	0.25	1.04 (0.97 to 1.12)	0.31
*Planned cesarean section*	1,794 (8.1)	5,541 (7.4)	**1.11 (1.05 to 1.18)**	**<0.001**	**1.08 (1.02 to 1.15)**	**0.01**	1.06 (1.00 to 1.13)	0.06
*Unplanned cesarean section*	1,969 (8.9)	5,697 (7.6)	**1.19 (1.13 to 1.26)**	**<0.001**	**1.12 (1.05 to 1.19)**	**<0.001**	**1.11 (1.04 to 1.18)**	**0.001**
*Unknown or missing*	7	58						
**The 1-min Apgar score**								
*≥7*	21,253 (96.0)	71,853 (96.3)	Reference	Ref	Reference	Ref	NA	
* <7*	891 (4.0)	2,751 (3.7)	**1.10 (1.02 to 1.19)**	**0.01**	1.04 (0.97 to 1.13)	0.40	NA	
*Missing*	37	122						
**Umbilical artery pH**								
Categorized model								
*≥7.15*	9,342 (91.3)	28,041 (91.6)	Reference	Ref	NA		NA	
* <7.15*	890 (8.7)	2,563 (8.4)	1.08 (0.98 to 1.19)	0.11	NA		NA	
*Missing*	11,949	44,122						
Continuous model (Mean 7.27, SD 0.09)			0.80 (0.58 to 1.09)	0.15	NA		NA	
**Neonatal monitoring**								
*No*	20,258 (91.3)	69,111 (92.5)	Reference	Ref	Reference	Ref	NA	
*Yes*	1,923 (8.7)	5,615 (7.5)	**1.18 (1.12 to 1.24)**	**<0.001**	1.02 (0.95 to 1.09)	0.60	NA	
*Missing*	0	0						

*CI, confidence interval; OR, odds ratio; aOR, adjusted odds ratio. Adjusted model 1: adjusted for mode of delivery, Apgar score, neonatal monitoring, maternal age, paternal age, number of previous deliveries, maternal psychiatric history, maternal smoking, maternal socioeconomic status, gestational age, weight for gestational age. Adjusted model 2: adjusted for mode of delivery, Apgar score, neonatal monitoring, maternal age, paternal age, number of previous deliveries, maternal psychiatric history, maternal smoking, maternal socioeconomic status, gestational age, weight for gestational age, maternal hypertensive disorders, maternal diabetes, maternal fear of giving birth. The adjusted model 2 was created only for mode of delivery. Bold values are statistically significant results*.

**Figure 2:**
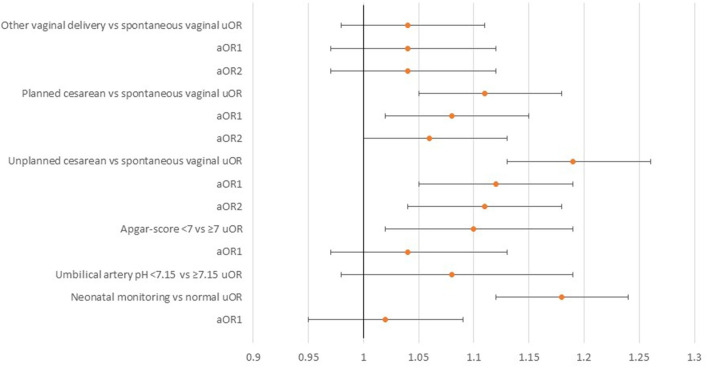
The associations between mode of delivery, birth outcomes, and anxiety disorders.

Table 1 and Figure 2 show the unadjusted and adjusted data. All exposures, but umbilical artery pH, were associated with increased odds for anxiety disorders and therefore included in the adjusted model. Umbilical artery pH was excluded because it was unavailable for 56.9% of the sample and showed no associations in the categorized or continuous unadjusted models. Offspring born by planned and unplanned cesarean sections were more likely to have anxiety disorders, with adjusted ORs (aORs) of 1.08 (95% CI 1.02–1.15) and 1.12 (95% CI 1.05–1.19), respectively. No associations were observed for any vaginal birth categories, Apgar score and neonatal monitoring.

Table 2A shows the frequencies of each exposure in the specific anxiety disorder groups and Table 2B shows the associations between the exposures and specific anxiety disorders in the unadjusted and adjusted models. Several associations emerged in the unadjusted analyses. Birth by planned cesarean sections were associated with increased odds for generalized anxiety disorder, social phobia and specific phobia. Birth by unplanned cesarean sections were associated with increased odds for generalized anxiety disorder, separation anxiety disorder, social phobia and specific phobia. Low Apgar scores were associated with increased odds for specific phobia and neonatal monitoring was associated with increased odds for elective mutism, separation anxiety disorder and specific phobia. No associations were found for umbilical artery pH. In the adjusted analyses, birth by planned cesarean section and neonatal monitoring were only associated with increased odds for specific phobias, aOR 1.24 (95% CI 1.04–1.48), and aOR 1.28 (95% CI 1.07–1.52), respectively.

**Table 2A:** Frequencies of the exposure variables in the cases with specific anxiety disorders and the controls.

**Exposure**	**Elective mutism** ** Cases *n* (%)** ** (*n =* 748)** ** Controls *n* (%)** ** (*n =* 2,532)**	**Generalized anxiety disorder** ** Cases *n* (%)** ** (*n =* 2,115)** ** Controls *n* (%)** ** (*n =* 7,160)**	**Panic disorder/ agoraphobia** ** Cases *n* (%)** ** (*n =* 1,939)** ** Controls *n* (%)** ** (*n =* 6,506)**	**Separation anxiety disorder** ** Cases *n* (%)** ** (*n =* 655)** ** Controls *n* (%)** ** (*n =* 2,185)**	**Social phobia** ** Cases *n* (%)** ** (*n =* 1,921)** ** Controls *n* (%)** ** (*n =* 6,428)**	**Specific phobia** ** Cases *n* (%)** ** (*n =* 2,567)** ** Controls *n* (%)** ** (*n =* 8,665)**
**Mode of delivery**						
*Spontaneous vaginal cephalic*	568 (75.9) 1,975 (78.1)	1,591 (75.3) 5,628 (78.6)	1,539 (79.4) 5,222 (80.3)	491 (75.0) 1,714 (78.6)	1,495 (77.9) 5,198 (80.9)	1,911 (74.4) 6,789 (78.4)
*Vaginal other*	50 (6.7) 174 (6.9)	128 (6.1) 450 (6.3)	101 (5.2) 336 (5.2)	41 (6.3) 126 (5.8)	108 (5.6) 339 (5.3)	171 (6.7) 541 (6.3)
*Planned cesarean section*	60 (8.0) 178 (7.0)	187 (8.9) 544 (7.6)	156 (8.1) 489 (7.5)	57 (8.7) 186 (8.5)	149 (7.8) 423 (6.6)	234 (9.1) 622 (7.2)
*Unplanned cesarean section*	70 (9.4) 201 (8.0)	207 (9.8) 535 (7.5)	142 (7.3) 457 (7.0)	66 (10.1) 156 (7.2)	167 (8.7) 462 (7.2)	251 (9.8) 707 (8.2)
*Missing*	0 4	2 3	1 2	0 3	2 6	0 6
**The 1-min Apgar score**						
*≥7*	713 (95.6) 2,419 (95.7)	2,038 (96.5) 6,879 (96.3)	1,860 (96.2) 6,280 (96.7)	621 (95.0) 2,101 (96.4)	1,843 (96.0) 6,194 (96.5)	2,440 (95.2) 8,323 (96.3)
***<**7*	33 (4.4) 110 (4.4)	75 (3.6) 266 (3.7)	74 (3.8) 217 (3.3)	33 (5.1) 78 (3.6)	77 (4.0) 224 (3.5)	122 (4.8) 323 (3.7)
*Missing*	2 3	2 15	5 9	1 6	1 10	5 19
**Umbilical artery pH**						
*≥7.15*	299 (90.1) 1,087 (91.9)	947 (92.0) 2,762 (91.6)	635 (92.8) 2,171 (93.2)	311 (91.2) 889 (91.6)	752 (91.4) 2,183 (92.2)	1,209 (90.0) 3,591 (90.3)
* <7.15*	33 (9.9) 96 (8.1)	82 (8.0) 252 (8.4)	49 (7.2) 159 (6.8)	30 (8.8) 82 (8.4)	71 (8.6) 184 (7.8)	134 (10.0) 384 (9.7)
*Missing*	416 1,349	1,086 4,146	1,255 4,176	314 1,214	1,098 4,061	1,224 4,690
**Neonatal monitoring**						
*No*	663 (88.6) 2,328 (91.9)	1,938 (91.6) 6,623 (92.5)	1,804 (93.0) 6,089 (93.6)	593 (90.5) 2,033 (93.0)	1,793 (93.3) 6,005 (93.4)	2,264 (88.2) 7,962 (91.9)
*Yes*	85 (11.4) 204 (8.1)	177 (8.4) 537 (7.5)	135 (7.0) 417 (6.4)	62 (9.5) 152 (7.0)	128 (6.7) 423 (6.6)	303 (11.8) 703 (8.1)
*Missing*	0 0	0 0	0 0	0 0	0 0	0 0

**Table 2B:** ORs (95% CIs) for the associations between the exposures and specific anxiety disorders in the unadjusted and adjusted analyses.

**Exposure**	**Elective mutism** ** Cases and controls in the adjusted analysis 704/2,435**	**Generalized anxiety disorder** ** Cases and controls in the adjusted analysis 2,007/6,925**	**Panic disorder/ agoraphobia** ** Cases and controls in the adjusted analysis 1,862/6,314**	**Separation anxiety ** ** Cases and controls in the adjusted analysis 629/2,098**	**Social phobia** ** Cases and controls in the adjusted analysis 1,844/6,220**	**Specific phobia ** ** Cases and controls in the adjusted analysis 2,465/8,365**
**Mode of delivery**			
*Vaginal other vs vaginal spontaneous*	uOR 1.01 (0.73 to 1.41) aOR^1^ 1.03 (0.70 to 1.50)	uOR 1.04 (0.85 to 1.28) aOR^1^ 0.96 (0.76 to 1.21)	uOR 1.02 (0.81 to 1.28) aOR^1^ 1.11 (0.86 to 1.42)	uOR 1.17 (081.to 1.69) aOR^1^ 1.06 (0.70 to 1.59)	uOR 1.13 (0.90 to 1.41) aOR^1^ 1.17 (0.91 to 1.49)	uOR 1.12 (0.93 to 1.34) aOR^1^ 1.08 (0.89 to 1.32) aOR^2^ 1.07 (0.88 to 1.31)
*Planned cesarean section vs vaginal spontaneous*	uOR 1.16 (0.85 to 1.59) aOR^1^ 0.97 (0.69 to 1.36)	uOR **1.23** **(1.03 to 1.46)** aOR^1^ 1.15 (0.94 to 1.40)	uOR 1.08 (0.89 to 1.31) aOR^1^ 1.04 (0.85 to 1.27)	uOR 1.06 (0.77 to 1.46) aOR^1^ 0.90 (0.63 to 1.29)	uOR **1.22** **(1.00 to 1.49)** aOR^1^ 1.14 (0.92 to 1.40)	uOR **1.36** **(1.16 to 1.59)** aOR^1^ **1.24** **(1.04 to 1.48)** aOR^2^ **1.21** **(1.01 to 1.44)**
*Unplanned cesarean section vs vaginal spontaneous*	uOR 1.24 (0.93 to 1.65) aOR^1^ 1.08 (0.77 to 1.51)	uOR **1.37** **(1.15 to 1.62)** aOR^1^ 1.17 (0.96 to 1.43)	uOR 1.06 (0.87 to 1.30) aOR^1^ 1.09 (0.88 to 1.36)	uOR **1.05** **(1.07 to 1.98)** aOR^1^ 1.23 (0.86 to 1.77)	uOR **1.25** **(1.04 to 1.51)** aOR^1^ 1.20 (0.98 to 1.48)	uOR **1.28** **(1.09 to 1.49)** aOR^1^ 1.17 (0.98 to 1.39) aOR^2^ 1.15 (0.97 to 1.37)
**The one-minute apgar score**						
* <7 vs ≥7*	uOR 1.03 (0.69 to 1.55) aOR^1^ 0.89 (0.56 to 1.40)	uOR 0.98 (0.75 to 1.27) aOR^1^ 0.90 (0.67 to 1.22)	uOR 1.17 (0.89 to 1.54) aOR^1^ 1.22 (0.90 to 1.65)	uOR 1.45 (0.95 to 2.19) aOR^1^ 1.36 (0.84 to 2.19)	uOR 1.16 (0.89 to 1.52) aOR^1^ 1.14 (0.85 to 1.53)	uOR **1.29** **(1.04 to 1.60)** aOR^1^ 1.01 (0.79 to 1.28)
**Umbilical artery pH**						
* <7.15 vs ≥7.15*	uOR 0.91 (0.55 to 1.50) aOR^1^ NA	uOR 0.93 (0.68 to 1.26) aOR^1^ NA	uOR 0.88 (0.55 to 1.39) aOR^1^ NA	uOR 1.02 (0.63 to 1.68) aOR^1^ NA	uOR 1.09 (0.75 to 1.58) aOR^1^ NA	uOR 1.08 (0.85 to 1.38) aOR^1^ NA
**Neonatal monitoring**						
*Yes vs No*	uOR **1.48** **(1.13 to 1.93)** aOR^1^ 1.32 (0.95 to 1.83)	uOR 1.12 (0.94 to 1.34) aOR^1^ 0.89 (0.71 to 1.11)	uOR 1.11 (0.91 to 1.36) aOR^1^ 0.94 (0.74 to 1.20)	uOR **1.47** **(1.07 to 2.01)** aOR^1^ 1.35 (0.92 to 1.99)	uOR 1.03 (0.84 to 1.26) aOR^1^ 0.95 (0.75 to 1.20)	uOR **1.52** **(1.32 to 1.76)** aOR^1^ **1.28** **(1.07 to 1.52)**

*CI, confidence interval; uOR, unadjusted odds ratio; aOR, adjusted odds ratio; aOR^1^, adjusted for mode of delivery, Apgar score, neonatal monitoring, maternal age, paternal age, number of previous deliveries, maternal psychiatric history, maternal smoking, maternal socioeconomic status, gestational age, weight for gestational age; aOR^2^, adjusted for mode of delivery, Apgar score, neonatal monitoring, maternal age, paternal age, number of previous deliveries, maternal psychiatric history, maternal smoking, maternal socioeconomic status, gestational age, weight for gestational age, maternal hypertensive disorders, maternal diabetes, maternal fear of giving birth. The adjusted model 2 was created only for mode of delivery and specific phobia. Bold values are statistically significant results*.

An additional conditional logistic regression model was created for delivery mode, with further adjustments (Tables 1, 2B). Birth by unplanned cesarean section was associated with increased odds for anxiety disorders, aOR 1.11 (95% CI 1.04–1.18) and planned cesarean section with increased odds for specific phobia, aOR 1.21 (95% CI 1.01–1.44). The association between birth by planned cesarean section and anxiety disorders became insignificant in the adjusted analyses, but showed a tendency for statistical significance, aOR 1.06 (95% CI 1.00–1.13).

## Discussion

This was the largest study to examine whether mode of delivery and birth outcomes were associated with anxiety disorders in childhood and adolescence and the first study to use national register data to identify associations with specific anxiety disorders. Children born by cesarean section were more likely to be diagnosed with child and adolescent anxiety disorders and specific phobias were more common if cesarean section was planned and neonatal monitoring was required after delivery.

Birth by planned and unplanned cesarean section showed increased odds for later anxiety disorders in the adjusted model. However, when maternal diagnoses of hypertensive disorders, diabetes and fear of giving birth were included, the association between birth by unplanned cesarean section and anxiety disorders remained, but birth by planned cesarean section only showed a tendency. The continuous increase in cesarean sections highlight the importance of these findings (33). Previous register-based studies that reported associations between cesarean sections and anxiety disorders later in life did not consider planned and unplanned cesarean sections separately and include comprehensive cofounders, such as indications for cesarean sections (14–16). A Swedish register-based study showed no such associations after full adjustment, unlike our study (13). These differences in the findings may be due to the definitions of anxiety disorders that were used. For example, Zhang et al. (13) included stress-related disorders, but not childhood onset disorders. In contrast, our study concentrated more precisely on child and adolescent anxiety disorders. Furthermore, Zhang et al. (13) included numerous perinatal confounders for cesarean sections (Supplementary Table 1) and the adjustment for maternal somatic problems, amniotic fluid and placental disorders, pelvic disproportion, malpresentation, dystocia, failed induction and fetal distress made their associations insignificant. We only included such confounders that were previously shown to be associated with the studied exposures and anxiety disorders (5–7, 15–17, 26–31). Although we did not include all the possible perinatal adversities, we did include indicators for fetal distress by studying Apgar scores and umbilical artery pH.

Several factors could explain the associations with cesarean sections. These include immunological mechanisms that influence neuronal development, prenatal or perinatal complications and maternal emotional factors. In animal models gut microbiome has related to altered neurodevelopment and emotional behavior (34). Similar mechanisms could contribute to anxiety disorders in humans. Immunological changes due to the disrupted transfer of microbiota from mother to child are likely to occur in cesarean sections (35). The microbiome can also be altered by antibiotics (36), which are typically administered during cesarean sections. Both planned and unplanned cesarean sections can follow prenatal or perinatal complications, such as pre-eclampsia, diabetes, fetal distress or infections (37) and these prenatal factors could contribute to the development of anxiety disorders (13, 16, 28, 30). Mothers who undergo cesarean sections may experience emotional distress and perceived loss of control and this can have impact their attachment to their child and parental behavior (38).

When we analyzed specific anxiety disorders, planned cesarean sections were associated with increased odds of specific phobias, but unplanned cesarean section was not. Studies have reported that women with psychiatric disorders are more likely to ask for cesarean sections (39). Maternal request for cesarean section can be derived from various fearful or anxious concepts (31). Phobic disorders run in families, with genetics playing a notable role (40). Our study was controlled for maternal psychiatric disorders diagnosed by specialized services and fear of giving birth. However, mothers with undiagnosed anxiety and less severe phobias could have contributed to the findings. Although we adjusted for the most common perinatal conditions, some residual confounding factors may have existed. Residual confounding factors have been suggested as plausible explanations for the associations between birth by cesarean sections and neurodevelopmental disorders in offspring, as the associations were attenuated in sibling analyses (13, 41).

Low Apgar score was associated with increased odds ratios for any anxiety disorder and for specific phobia in the unadjusted analyses. However, no significant results emerged in the adjusted analyses. Previously, one study has examined the 1-min Apgar score and anxiety disorders with no association (15). For the 5-min Apgar score, a significant association for increased odds of child anxiety disorders was found in one study (17), but not in the other (16). Apgar score is an unspecific measurement of infant's color, heart rate, reflexes, muscle tone and respiration, and it is ideally used for describing newborn infant's well-being and resuscitation response (24). The measurement time, as well as the unspecific nature of the score, could contribute to the discrepancies in the findings. Low Apgar score does not seem to be independently associated with increased odds for anxiety disorders but the found associations are explained by other factors.

Children who needed neonatal monitoring were more likely to be diagnosed with specific phobias. A relatively small cohort study, found that NICU treatment was associated with specific phobia and separation anxiety in children, based on specific anxiety disorder diagnoses reported by the children and their parents (19). The association between neonatal monitoring and specific phobia could be explained by residual perinatal adversities, similar to those for cesarean sections, as discussed above. In addition, NICU treatment notably increases newborn stress (42). Some infants treated in NICU have severe conditions and hence require multiple, painful and frightening procedures even after they are discharged. These potentially traumatizing experiences could predispose these children to later specific phobia (43). Painful NICU procedures have been associated with alterations in early brain development (44) and with stress responses (45). Newborn stress may also be increased by parental separation, as fewer parental NICU visits have been associated with later behavioral problems in offspring (46). Parental NICU visits have only increased in recent decades (47). Furthermore, NICU treatment increases parental stress, which could have an impact on parent-child interactions (12) and attachment (7). Neonatal adversities may also lead to parents being more sensitive to their child's conditions and problems (48).

Certain limitations should be considered. The anxiety disorder diagnoses were from specialized healthcare and less severe anxiety disorders were not included. Umbilical artery pH was only available for less than half the sample, which limited its use, and may have contained incorrect recordings, such as venous samples reported as arterial values. The ICD-10 diagnostic code for fear of giving birth was only added in in 1997 (23, 32) and was not available for the oldest subjects. The 1-min Apgar score was well recorded. The 5-min score would have been preferable, but it was only available for 2.9% of the sample. The specific anxiety disorder groups were relatively small, as most diagnoses were non-specific anxiety disorders. The anxiety disorder diagnoses had not been validated, except for elective mutism (49). However, the psychiatric diagnoses in the Health Care register had been validated as accurate (7, 50). We were unable to address all the possible residual confounders, as the data in the registers was not comprehensive for all perinatal events. We did not have data on the later life events for cases and controls, such as childhood adversities, which could contribute to the development of anxiety disorders. Globally varying rates of cesarean sections and focusing on singleton pregnancies may influence the generalizability of the results.

## Conclusion

Children and adolescents born by cesarean section were more likely to have anxiety disorders later in childhood and adolescence, especially if cesarean section was unplanned. However, we were unable to show whether these associations were affected by residual confounding factors, such as additional perinatal adversities or maternal stress and anxiety. The constantly increasing rate of cesarean sections underlines the importance of our results.

## Data Availability Statement

The datasets presented in this article are not readily available because the data are available on Finnish Social and Health Data Permit Authority (FINDATA) and were used under a license for this study. Data are available from the authors upon reasonable request and with permission of FINDATA. Requests to access the datasets should be directed to info@findata.fi, tthuht@utu.fi.

## Author Contributions

TS drafted the initial manuscript and contributed to the study design, data analysis, data interpretation, and literature search. SU and PP-K contributed to the study design, data analysis, data interpretation, and reviewed and revised the manuscript. PK and TL contributed to the study design, data interpretation, and reviewed and revised the manuscript. SH-Y-S contributed to the study design, conducted the statistical analyses, and reviewed and revised the manuscript. AS conceptualized the study, contributed to the study design, data collection and data interpretation, organized the funding of the study, and reviewed and revised the manuscript. All authors approved the final manuscript as submitted and agree to be accountable for all aspects of the work.

## Funding

AS received funding from the Academy of Finland Flagship Programme (decision number 320162), the Strategic Research Council at the Academy of Finland (decision number 303581), and the Academy of Finland Health from Cohorts and Biobanks Programme (decision number 308552). TS received funding from Finnish Brain Foundation. The funders played no role in any aspect of the study or manuscript.

## Conflict of Interest

The authors declare that the research was conducted in the absence of any commercial or financial relationships that could be construed as a potential conflict of interest.

## Publisher's Note

All claims expressed in this article are solely those of the authors and do not necessarily represent those of their affiliated organizations, or those of the publisher, the editors and the reviewers. Any product that may be evaluated in this article, or claim that may be made by its manufacturer, is not guaranteed or endorsed by the publisher.
