# Supplementary material for: Associations Between Delivery Modes, Birth Outcomes and Offspring Anxiety Disorders in a Population-Based Birth Cohort of Children and Adolescents
Source: Front Psychiatry. 2022 Jul 13;13:917299. doi: 10.3389/fpsyt.2022.917299 (PMC9326080; doi:10.3389/fpsyt.2022.917299)
Supplement: Supplementary file 1 [file Data_Sheet_1.pdf]

Associations between delivery modes, birth outcomes and offspring anxiety disorders in a population-based birth cohort of children and adolescents

Supplementary data

Table S1. Previous register-based studies on delivery outcomes and anxiety disorders.

Table S2. Frequencies of exposure variables and confounders in cases and controls. Associations between the confounders and the exposure variables and between the confounders and the outcome variables.

Table S1. Previous register-based studies on delivery outcomes and anxiety disorders.

Study	Total sample n<br><br>(anxiety disorders n)	Age range	Exposures	Outcomes	Confounders	Results
Chen, X., Kong, L., Piltonen, T. T., Gissler, M., & Lavebratt, C. (2020). Association of polycystic ovary syndrome or anovulatory infertility with offspring psychiatric and mild neurodevelopmental disorders: a Finnish population-based cohort study. *Human Reproduction*, *35*(10), 2336-2347.<br><br>Finland	1 097,753<br><br>(24,746)	4 to 22	Polycystic ovary syndrome. Additional analyses included cesarean sections.	F40–F43 and F93	Maternal age, parity, immigrant status, marital status, smoking, maternal purchase of psychotropic drugs during pregnancy and maternal systemic inflammatory disease.	**No polycystic ovary syndrome and cesarean section HR 1.13 (95% CI 1.10–1.15)**
Guhn, M., Emerson, S. D., Mahdaviani, D., & Gadermann, A. M. (2020). Associations of birth factors and socio-economic status with indicators of early emotional development and mental health in childhood: a population-based linkage study. *Child Psychiatry & Human Development*, *51*(1), 80-93.<br><br>Canada	89,404<br><br>(7,867)	5 to 15	Multiple exposures studied. We were interested in: cesarean sections, forceps and breech deliveries and one-minute Apgar scores.	F40-F43	All the exposures were studied in the same multivariate model: sex, mode of delivery, birth weight, gestational age, Apgar scores, maternal age, immigrant status, marital status and maternal socioeconomic status.	**Cesarean section OR 1.12 (95% CI 1.06 - 1.19).** Forceps and breech deliveries NS. The one-minute Apgar scores NS.
Kingston, D., Heaman, M., Brownell, M., & Ekuma, O. (2015). Predictors of childhood anxiety: a population-based cohort study. *PLoS One*, *10*(7), e0129339.<br><br>Canada	18,836<br><br>(591)	≤5	Multiple exposures studied. We were interested in: five-minute Apgar scores and cesarean sections.	F40-F43, F93, prescription of medication, ICD-9-CM code 300, 300.2, 300.3	All the exposures were studied in the same multivariate model: maternal age, education, income, relationship status, parity, type of delivery, antepartum hemorrhage, social isolation, relationship distress, psychological distress, diabetes, hypertension, substance use, infant sex, Apgar score, gestational age, small for gestational age, breastfeeding initiation.	**The five-minute Apgar score ≤ 7 OR 1.76 (95% CI 1.20 – 2.58).** Cesarean section NS

Larsen, J. T., Bulik, C. M., Thornton, L. M., Koch, S. V., & Petersen, L. (2021). Prenatal and perinatal factors and risk of eating disorders. *Psychological medicine*, *51*(5), 870-880.<br><br>Denmark	1 167,043<br><br>(15,205)	6 to 27	Multiple exposures studied. We were interested in: five-minute Apgar scores and cesarean sections.	F40, F41	Sex, age, calendar-time and maternal history of the same psychiatric disorder	**cesarean section HR 1.06 (95% CI 1.01-1.11).** The five-minute Apgar NS.
Zhang, T., Brander, G., Mantel, Ä., Kuja-Halkola, R., Stephansson, O., Chang, Z., ... & de la Cruz, L. F. (2021). Assessment of cesarean delivery and neurodevelopmental and psychiatric disorders in the children of a population-based Swedish birth cohort. *JAMA network open*, *4*(3), e210837-e210837.<br><br>Sweden	1 179,341<br><br>(41,929)	6 to 23	Cesarean sections.	F40, F41, F43.<br><br>ICD-9: 300A, 300C, 308, 309	Model 1: sex and year of birth.<br>Model 2: sex, year of birth, gestational age, parental age, parity, maternal education, smoking and maternal and paternal history of psychiatric disorders.<br>Model 3: Further adjusted for maternal hypertension, diabetes, infections during pregnancy, fetal malpresentation, large for gestational age, polyhydramnios, oligohydramnios and preeclampsia.<br>For planned cesarean sections: further adjusted for pelvic disproportion.<br>For unplanned caesarean sections: further adjusted for pelvic disproportion, placental disorders, dystocia, failed induction, and fetal distress.	**Planned cesarean section Model 2 HR 1.10 (95% CI 1.05-1.16);** Model 3 HR 1.07 (95% CI 1.00-1.14). **Unplanned cesarean section Model 2 HR 1.08 (95% CI 1.03-1.13);** Model 3 HR 1.02 (95% CI 0.96-1.09)

Bold values represent statistically significant results. CI = confidence interval; HR = hazard ratio; NS = not significant; OR = odds ratio

Table S2. Frequencies of exposure variables and confounders in cases and controls. Associations between the confounders and the exposure variables and between the confounders and the outcome variables.

**Exposure/confounder**	**Cases n (%) 22,181**	**Controls n (%) 74,726**	**Missing values**	**Association with exposure** Mode of delivery/ Apgar score/ Umbilical artery pH/ Neonatal monitoring	**Association with outcome** Any anxiety disorder
**Mode of delivery**			65 (0.1)	NA; **<0.001**; **<0.001**; **<0.001**	**See Table 1**
Spontaneous vaginal cephalic	17,132 (77.3)	59,147 (79.2)			
Vaginal other	1,279 (5.8)	4,283 (5.7)			
Planned cesarean section	1,794 (8.1)	5,541 (7.4)			
Unplanned cesarean section	1,969 (8.9)	5,697 (7.6)			
**The one-minute Apgar score**			159 (0.2)	**<0.001**; NA; **<0.001**; **<0.001**	**See Table 1**
≥7	21,253 (96.0)	71,853 (96.3)			
<7	891 (4.0)	2,751 (3.7)			
**Umbilical artery pH**			56,071 (57.9)	**<0.001**; **<0.001**; NA; **<0.001**	**See Table 1**
≥7.15	9,342 (91.3)	28,041 (91.6)			
<7.15	890 (8.7)	2,563 (8.4)			
**Neonatal monitoring (maternal postpartum ward or neonatal intensive care unit)**			0	**<0.001**; **<0.001**; **<0.001;** NA	**See Table 1**
No	20,258 (91.3)	69,111 (92.5)			
Yes	1,923 (8.7)	5,615 (7.5)			
**Maternal age**				**<0.001**; **0.04**; **<0.001**; **<0.001**	**<0.001**
≤19	1,032 (4.7)	1,879 (2.5)			
20-29	11,587 (52.2)	38,743 (51.9)			
30-39	8,775 (39.6)	32,056 (42.9)			
≥40	787 (3.6)	2,048 (2.7)			
**Paternal age**			1,144	**<0.001;** 0.37; **<0.001; <0.001**	**<0.001**
≤19	321 (1.5)	482 (0.7)			
20-29	8704 (40.2)	28,675 (38.7)			
30-39	10,170 (46.9)	37,940 (51.2)			
≥40	2,478 (11.4)	6,993 (9.4)			
**Number of previous births**			66	**<0.001**; **<0.001**; **<0.001**; **<0.001**	**<0.001**
0	9,548 (43.1)	29,692 (39.8)			
≤1	12,614 (56.9)	44,987 (60.2)			
**Maternal psychiatric history (substance abuse disorders excluded)**			0	**<0.001**; 0.47; 0.15; **<0.001**	**<0.001**
Yes	7,625 (34.4)	10,684 (14.3)			
No	14,556 (65.6)	64,042 (85.7)			

**Maternal substance abuse**			0	0.11; 0.07; 0.75; **<0.001**	**<0.001**
Yes	1,878 (8.5)	1,890 (2.5)			
No	20,303 (91.5)	72,836 (97.5)			
**Maternal smoking** (no collinearity was observed between maternal SES and smoking)			2,110	**0.006**; 0.33; 0.34; **0.005**	**<0.001**
Yes	5,481 (25.3)	11,064 (15.1)			
No	16,208 (74.7)	62,044 (84.9)			
**Maternal SES** (no collinearity was observed between maternal SES and smoking)				**<0.001**; 0.12; 0.89; **0.006**	**<0.001**
Upper white-collar workers	2,700 (12.2)	11,516 (15.4)			
Lower white-collar workers	8,957 (40.4)	33,667 (45.1)			
Blue-collar workers	4,712 (21.2)	13,817 (18.5)			
Others	4,523 (20.4)	12,374 (16.6)			
Missing	1,289 (5.8)	3,352 (4.5)			
**Gestational age**			0	**<0.001**; **<0.001**; **<0.001**; **<0.001**	**<0.001**
≤31 weeks	160 (0.7)	358 (0.5)			
32 - 36	965 (4.4)	2,756 (3.7)			
37 - 41	19,978 (90.1)	68,194 (91.3)			
≥ 42	1,078 (4.9)	3,418 (4.6)			
**Weight for gestational age**			0	**<0.001**; **<0.001**; 0.09; **<0.001**	**<0.001**
< -2SD	873 (3.9)	2,087 (2.8)			
-2SD - +2SD	20,570 (92.7)	69,915 (93.6)			
>2SD	738 (3.3)	2,724 (3.6)			
**Maternal hypertension pre-eclampsia or eclampsia**			58	**<0.001**; NA; NA; NA	**<0.001**
Yes	948 (4.3)	2,845 (3.8)			
No	21,233 (95.7)	71,881 (96.2)			
**Maternal diabetes**			58	**<0.001**; NA; NA; NA	**<0.001**
Yes	1,230 (5.5)	3,237 (4.3)			
No	20,951 (94.5)	71,489 (95.7)			
**Fear of giving birth**			58	**<0.001**; NA; NA; NA	**<0.001**
Yes	218 (1.0)	412 (0.6)			
No	21,963 (99.0)	74,314 (99.4)			

Bold values represent statistically significant results. NA = not applicable; SES = socioeconomic status; SD = standard deviation.
